# Genomic characterization of three bacteriophages targeting multidrug resistant clinical isolates of *Escherichia, Klebsiella* and *Salmonella*

**DOI:** 10.1007/s00203-022-02948-0

**Published:** 2022-05-19

**Authors:** Roshan Nepal, Ghais Houtak, Sumeena Karki, Gunaraj Dhungana, Sarah Vreugde, Rajani Malla

**Affiliations:** 1 Faculty of Health and Medical Sciences, The University of Adelaide, Adelaide, Australia; 2 Department of Surgery-Otolaryngology Head and Neck Surgery, The Basil Hetzel Institute for Translational Health Research, Central Adelaide Local Health Network, South Australia, Australia; 3 Central Department of Biotechnology, Tribhuvan University, Kirtipur, Nepal

**Keywords:** Bacteriophage, Phage, Genomics, Phage therapy, *Enterobacteriaceae*

## Abstract

Application of bacteriophages (phages) to treat complex multidrug-resistant bacterial infection is gaining traction because of its efficacy and universal availability. However, as phages are specific to their host, a diverse collection of locally isolated phage from various geographical locations is required to formulate a wide host range phage cocktail. Here, we report morphological and genomic features of three newly isolated phages from river water of the urban region in Kathmandu, Nepal, targeting three different bacteria (*Escherichia coli, Klebsiella pneumoniae* and *Salmonella enterica.*) from the *Enterobacteriaceae* family. Morphological identification and genome analysis indicated that two phages (Escherichia phage vB_EcoM_TU01 and Klebsiella phage vB_KpnP_TU02) were strictly lytic and free from integrases, virulence factors, toxins and known antimicrobial resistance genes, whereas Salmonella phage vB_SalS_TU03 was possibly a temperate phage. The genomic features of these phages indicate that natural phages are capable of lysing pathogenic bacteria and may have potential in bacterial biocontrol.

**Supplementary Information:**

The online version contains supplementary material available at 10.1007/s00203-022-02948-0.

## Introduction

*Enterobacteriaceae* is a large family of Gram-negative rod-shaped facultatively anaerobic bacteria comprising a wide range of pathogens such as *Escherichia, Klebsiella*, *Salmonella*, *Enterobacter*, *Citrobacter*, *Shigella* and more. These pathogens are associated with considerable morbidity and mortality on compromised hosts and can cause life-threatening illnesses like septicaemia, haemolytic uremic syndrome, gastroenteritis, meningitis and pneumonia in healthy individuals (Donnenberg et al. 2015). These infections are usually treated with antibiotics, but lately, most human-associated pathogens are becoming increasingly resistant to antibiotics, thereby limiting the effectiveness of the antibiotic treatment. Furthermore, the emergence of carbapenem-resistant *Enterobacteriaceae* is a concern as there is no therapy or vaccines available to prevent acquisition of infection with multidrug resistant (MDR) strains. As current antibiotic therapies are ineffective to treat such infections or eliminate once infected, alternative approaches are highly sought in the management of MDR infections.

Bacteriophage (phage) is a virus that infects bacterial cells but leaves eukaryotes unscathed. Because of its host specificity, phages can be used to kill bacteria without harming untargeted cells. In the past decade, therapeutic application of phage has been gaining widespread attention because of its specificity and efficacy against MDR bacterial pathogens (Pirnay 2020). Further it is also regarded as ‘dynamic’ solution to continuously emerging MDR strains because of its co-evolving lifestyle with the bacteria. Phage therapy uses ‘strictly’ lytic phages or its derivatives to kill pathogenic bacteria. Although phage therapy is not novel and had been employed shortly after the discovery of phages around 1920s (d'Herelle 1931), invention of antibiotics curbed the widespread usage of phages therapeutically as antibiotics were more effective against a broad spectrum of bacteria. However, emergence of multidrug-resistant ‘superbugs’ has rekindled the interest in phage therapy. Studies have shown that phage therapy can be used as an alternative biocontrol agent or adjuvant therapy to antibiotics in human and animals (Petrovic Fabijan et al. 2020; Schooley et al. 2017; Ooi et al. 2019; Waters et al. 2017; Greene et al. 2021).

However, the efficacy of phage therapy targeting the pathogen of interest still has room for improvement. As phages are highly specific in regard to infecting their host, extending up to the level of bacterial strains, phages isolated from geographically same region as the bacterial host would have a higher probability of infecting the bacterial strain of interest due to the co-evolutionary adaptations (Hampton et al. 2020). Therefore, a local ‘phage bank’ comprising various phages isolated in the same region as bacterial pathogens of interest would facilitate a more effective strategy for the use of phages. Further, since most of the genes in phage genome is yet ‘hypothetical’, a comprehensive database reporting phage genome from different geolocations and clinical isolates is essential to study the co-evolution between phage and bacteria. As such, genome report provides invaluable information that can be useful in elucidating ‘conserved and unknown’ functions in phage genomes. Furthermore, the use of genomics and phenotyping of phages and their host could improve the efficacy of phage therapy in the future regarding the choice of phage for the pathogen of interest. In line with the aim of expanding phage research, previously, we reported phages exhibiting lytic activity against multidrug resistant *Pseudomonas* and *Klebsiella* (Dhungana et al. 2021a; Maharjan et al. 2022) and also studied pharmacokinetics and pharmacodynamics of our Klebsiella phage Kp_Pokalde_002 in a mouse model (Dhungana et al. 2021b). Here, we report the isolation, genome analysis and taxonomic position of three newly isolated phages targeting MDR human pathogens: *Escherichia coli*, *Klebsiella pneumoniae* and *Salmonella enterica* from *Enterobacteriaceae* family.

## Materials and method

### Bacterial strain

Three multidrug-resistant clinical isolates of *E. coli* (*N* = 1), *K. pneumoniae* (*N* = 1) and *S. enterica* (*N* = 1) were collected from the Microbiology Laboratory, Tribhuvan University Teaching Hospital, Kathmandu, Nepal. The clinical isolates were confirmed to be MDR by AMR testing in the microbiology department of the hospital and used as hosts for isolation and amplification of phages. The MDR status was also validated evaluating the strains against 11 different antibiotics (Supplementary table S1) using Kirby–Bauer disc-diffusion method (Hudzicki 2009). Nutrient agar (NA, agar = 1.5%, HiMedia, India) was used to grow fresh overnight culture (at 37 °C) from glycerol stock and Luria–Bertani broth (HiMedia, India) was used to propagate the host bacterium for phage isolation and amplification.

### Phage manipulation: isolation, purification and amplification

A water sample was collected from the Bagmati river, Kathmandu, Nepal flowing through the urban region of the city which is heavily polluted by untreated sewers and industrial waste (Mishra et al. 2017). Phages were isolated using Double Layer Agar Assay (DLAA) as described previously with some modifications (Dhungana et al. 2021a). Briefly, the water sample was centrifuged at 3220*g* (Centrifuge 5810R, Eppendorf, Hamburg, Germany) for 10 min to pellet down the debris and subsequently the supernatant was filtered through a 0.45-μm and 0.22-μm pore-size Whatman^™^ syringe filter (Sigma-Aldrich, Missouri, United States). One millilitre filtrate was mixed with 100 µl exponentially growing host bacteria (OD_600_ 0.5) and left at room temperature (10 min) for phage adsorption. Three millilitre semisolid top agar (Tryptic Soya Agar (TSA), agar = 0.4%, stored at = 50 °C) (HiMedia, India) was added to the mixture, mixed well by swirling and poured on to the surface of previously prepared bottom agar (TSA, agar = 1.0%, HiMedia, India). After overnight incubation at 37 °C, the plates were examined for the presence of phages in the form of plaques. A single isolated clear plaque was cut and dissolved in 1.0 mL of Sodium chloride-Magnesium sulfate (SM) buffer (10 mM Tris–HCl, 10 mM MgSO_4_.7H_2_O, 2% gelatin and 100 mM NaCl, pH 7.5). Subsequently, the phage was purified by performing three rounds of DLAA from a single isolated plaque.

### Phage characterization

#### Transmission electron microscopy

High titre purified phage lysates were fixed with fixative (2.5% glutaraldehyde and 2% paraformaldehyde prepared in 0.1 M sodium phosphate buffer (pH 7.2)). For fixation, equal volume of phage lysate and fixative were added, mixed and left overnight. The next day, the fixed phages were subjected to high-speed centrifugation (35,000*g*) for 3 h. Per sample 10.0 μL fixed phage lysate was deposited on a separate 300 mesh carbon-coated copper grid. The copper grid was then flooded with 2% (w/v) uranyl acetate (pH 4.5) for 2 min. The copper grid was dried and examined in JEM-2100F Transmission Electron Microscope (JEOL, USA) at 200 kV under different magnifications. TEM micrographs were processed using ImageJ 1.50i (https://imagej.nih.gov/ij) (Schneider et al. 2012).

#### Genomic DNA extraction, sequencing and annotation

Phage genomic DNA (gDNA) was isolated using Phage DNA Isolation Kit (Norgen Biotek Corp., Ontario, Canada. Cat. #46,800) per manufacturer’s instructions. Qualitative and quantitative control were performed using conventional electrophoresis and Qubit^*®*^ 2.0 Fluorometer (ThermoFisher Scientific, USA), respectively. Five microliter gDNA of each sample was loaded on 1% agarose gel and run for 30 min at 110 Volt. Also, 1.0 μl of each sample was loaded in NanoDrop 8000 (ThermoFisher Scientific, USA) for determining A260/280 ratio and Qubit^®^ 2.0 for determining concentration of gDNA.

The paired-end sequencing library was prepared using TruSeq^®^ Nano DNA HT Library Preparation Kit (Illumina, USA). Two hundred nanograms of gDNA was fragmented by Covaris shearing that generated dsDNA fragments with 3' or 5' overhangs. The fragments were then subjected to end-repair. The ligated products were purified using SP beads supplied in the kit. The size-selected product was PCR amplified as described in the protocol. The amplified library was analyzed in Bioanalyzer 2100 (Agilent Technologies, USA) using High Sensitivity (HS) DNA chip as per manufacturer's instructions. After obtaining the Qubit^®^ concentration for the library and the mean peak size from Bio-analyser profile (Fig. S1A–C), the library was loaded onto Illumina HiSeq 2000/2500 (Illumina, USA) for cluster generation and sequencing. The cluster generated was assembled using CLC Genomics Workbench 6.0 (Qiagen, USA) at default parameters (Minimum contig length: 200, Automatic word size: Yes, Perform scaffolding: Yes, Mismatch cost: 2, Insertion cost: 3, Deletion cost: 3, Length fraction: 0.5, Similarity fraction: 0.8). Phage genomes were annotated for coding DNA sequences (CDS), tRNA, virulence factors, toxins, antimicrobial resistance genes (ARGs) and drug targets using the Pathosystems Resource Integration Center (PATRIC 3.6.12) webtool (https://www.patricbrc.org/) (Wattam et al. 2013; Brettin et al. 2015) using viruses (taxid = 10,239) as the reference database. A circular map of the phage genome was generated using CGview server (http://cgview.ca/) (Stothard and Wishart 2004), and a phylogenetic tree was constructed BLASTing the query sequence against NCBI database using neighbor-joining method. Only the ten most common phages were included in the phylogenetic analysis. The tree was further visualized using ggtree package in R 4.1.1 (https://www.R-project.org/). The lifestyle, order, family and host of the phages were computationally predicted through PhageAI (https://phage.ai/) (Tynecki et al. 2020).

## Results and discussion

**Fig. 1:**
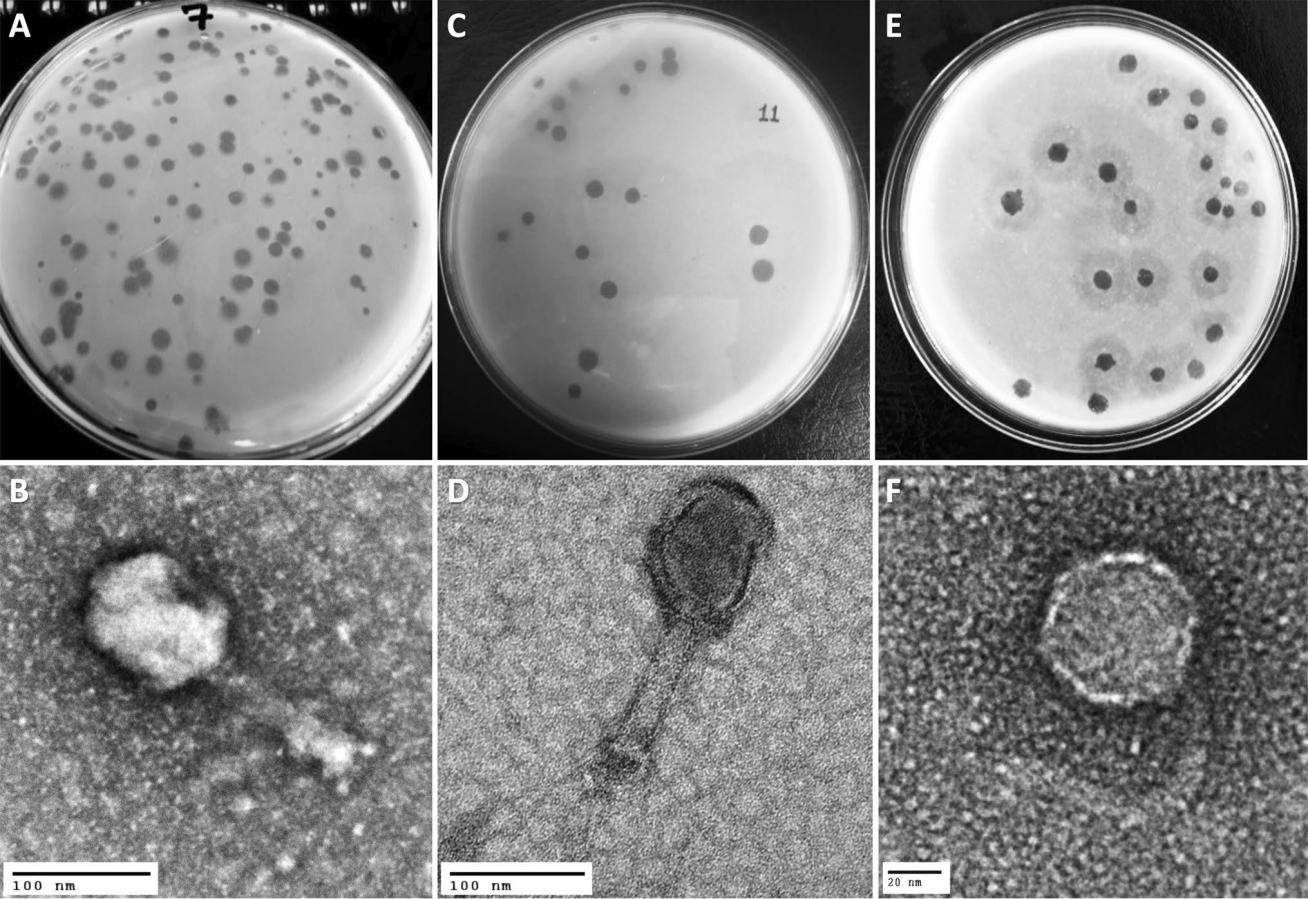
Phage isolation using double layer agar assay and their transmission electron micrograph (TEM). **A**, **C**, **E** Three double layered agar plates showing different types of phage plaque morphologies isolated directly from river water. **B** TEM of Escherichia phage vB_EcoM_TU01 (scale bar = 100 nm), **D** TEM of Klebsiella phage vB_KpnM_TU02 (scale bar = 100 nm), **F** TEM of Salmonella phage vB_SalS_TU03 (scale bar = 20 nm)

Three following phages, viz: Escherichia phage vB_EcoM_TU01 (hereafter vB_EcoM_TU01), Klebsiella phage vB_KpnM_TU02 (hereafter vB_KpnM_TU02) and Salmonella phage vB_SalS_TU03 (hereafter vB_SalS_TU03) targeting multidrug resistant clinical isolates of *E. coli*, *K. pneumoniae* and *S. enterica*. were isolated from the water sample collected from the Bagmati river (Fig. 1A, C, E). TEM revealed that among three phages, two (vB_EcoM_TU01, vB_KpnM_TU02) were from the *Myoviridae* family whereas vB_SalS_TU03 belonged to *Siphoviridae* family (Fig. 1B, D, F and Table 1). All phages were tailed phages (Order = *Caudovirales*) and consist of a linear double-stranded DNA (dsDNA) genome with gene density of approximately 1.7 genes/kilo-basepairs which is much higher that of the bacterial host (0.5–1.0 genes/kilo-basepairs) (Norwood and Sands 1997). The CDS coverage of all the phages was higher than 95% whereas the average gene length ranged between 540 and 567 basepairs (Table 2).

**Table 1:** Classification of phages according to ICTV* guidelines (ICTV 9th report) based on transmission electron micrograph

Phage	Capsid (in nm^)	Tail (W × L, in nm^)	Shape	Family (Morphotype^#^)
vB_EcoM_TU01,	82 × 108	19 × 111	Elongated	Myoviridae (A2)
vB_KpnM_TU02	82 × 99	25 × 109	Elongated	Myoviridae (A2)
vB_SalS_TU03	63	9 × 106	Icosahedral	Siphoviridae (B1)

*ICTV = The International Committee on Taxonomy of Viruses. ^ nm = nanometre. The capsid and tail lengths are an average of three measurements of a phage electron micrograph from a purified stock.

#Morphotypes are based on classification by Ackermann (2001)

**Table 2:** Genomic and protein features of three novel phages targeting multidrug resistant *Escherichia coli*, *Klebsiella pneumoniae* and *Salmonella enterica* clinical isolates

Features	Escherichia phage vB_EcoM_TU01	Klebsiella phage vB_KpnM_TU02	Salmonella phage vB_SalS_TU03
**NCBI accession**	**MZ560701**	**MZ560702**	**MZ560703**
**Genomic features**			
Length (in base pairs)	169,046 bp	166,230 bp	41,756 bp
Guanine-cytosine (G + C) content	37.42%	38.34%	47.06%
Total CDS	286	294	71
tRNAs	2	15	0
Gene density (per kbp)	1.69	1.77	1.70
Average gene size (in bp)	566	540	562
CDS coverage	95.9%	95.6%	95.7%
**Protein feature**			
Hypothetical proteins	48 (16.78%)	184 (62.59%)	26 (36.62%)
Functional proteins	238 (83.22%)	110 (37.41%)	45 (63.38%)
Proteins with GO assignments	16 (5.60%)	11 (3.74%)	2 (2.82%)
**Other features/genes**			
Transporter genes (Ref = TCDB)	5	0	0
Drug target genes (Ref = DrugBank)	3	0	0
Order	Caudovirales	Caudovirales	Caudovirales
Family	Myoviridae	Myoviridae	Siphoviridae
Genus (Ref = PhageAI, NCBI)	Mosigvirus	Jiaodavirus	Jerseyvirus
Lifestyle (Ref = PhageAI)	Virulent (C = 96%)	Virulent (C = 99%)	Temperate (C = 57%)

*NCBI*  National Center for Biotechnology Information, *CDS* Coding DNA sequences, *tRNA*  transfer RNA, *kbp* kilo basepairs, *GO*  Gene ontology (http://geneontology.org/), *TCDB*  Transporter classification database (https://www.tcdb.org/), *C*  Confidence

**Fig. 2:**
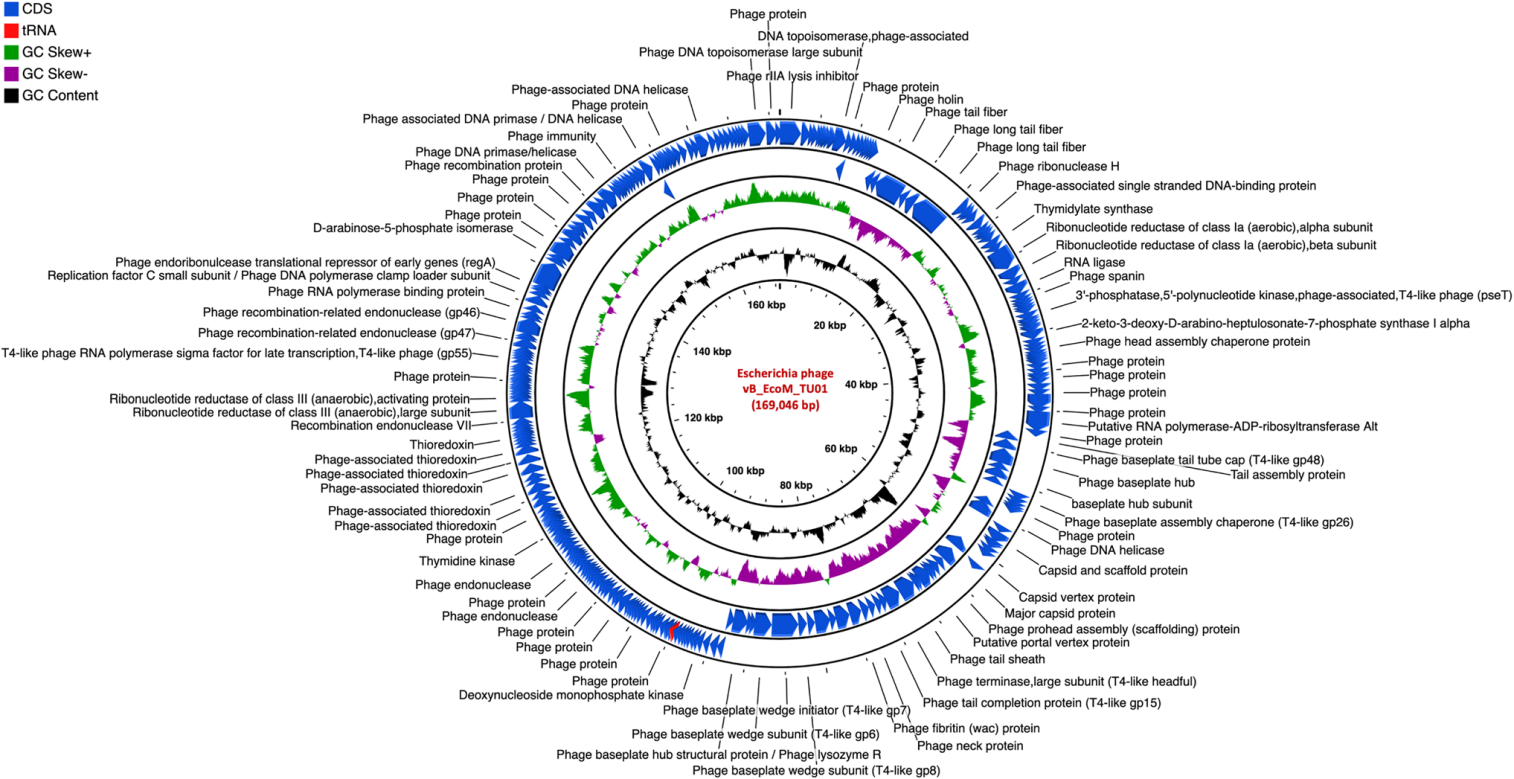
Genome organization of Escherichia phage vB_EcoM_TU01 targeting multidrug resistant *Escherichia coli* clinical isolate*.* Predicted coding regions are shown by arrows indicating the direction of the transcription

**Fig. 3:**
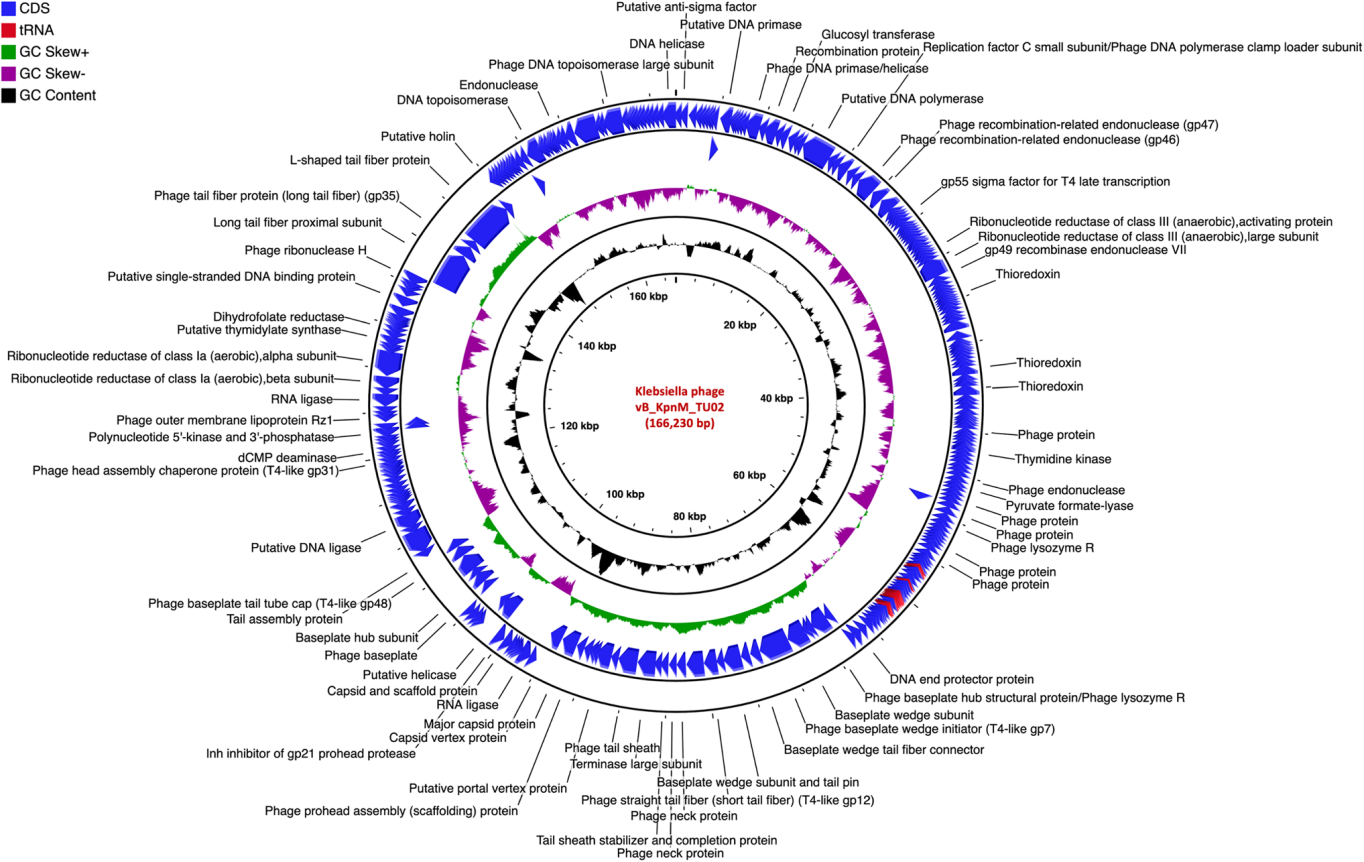
Genome organization of Klebsiella phage vB_KpnM_TU02 targeting multidrug resistant *Klebsiella pneumoniae* clinical isolate. Predicted coding regions are shown by arrows indicating the direction of the transcription

**Fig. 4:**
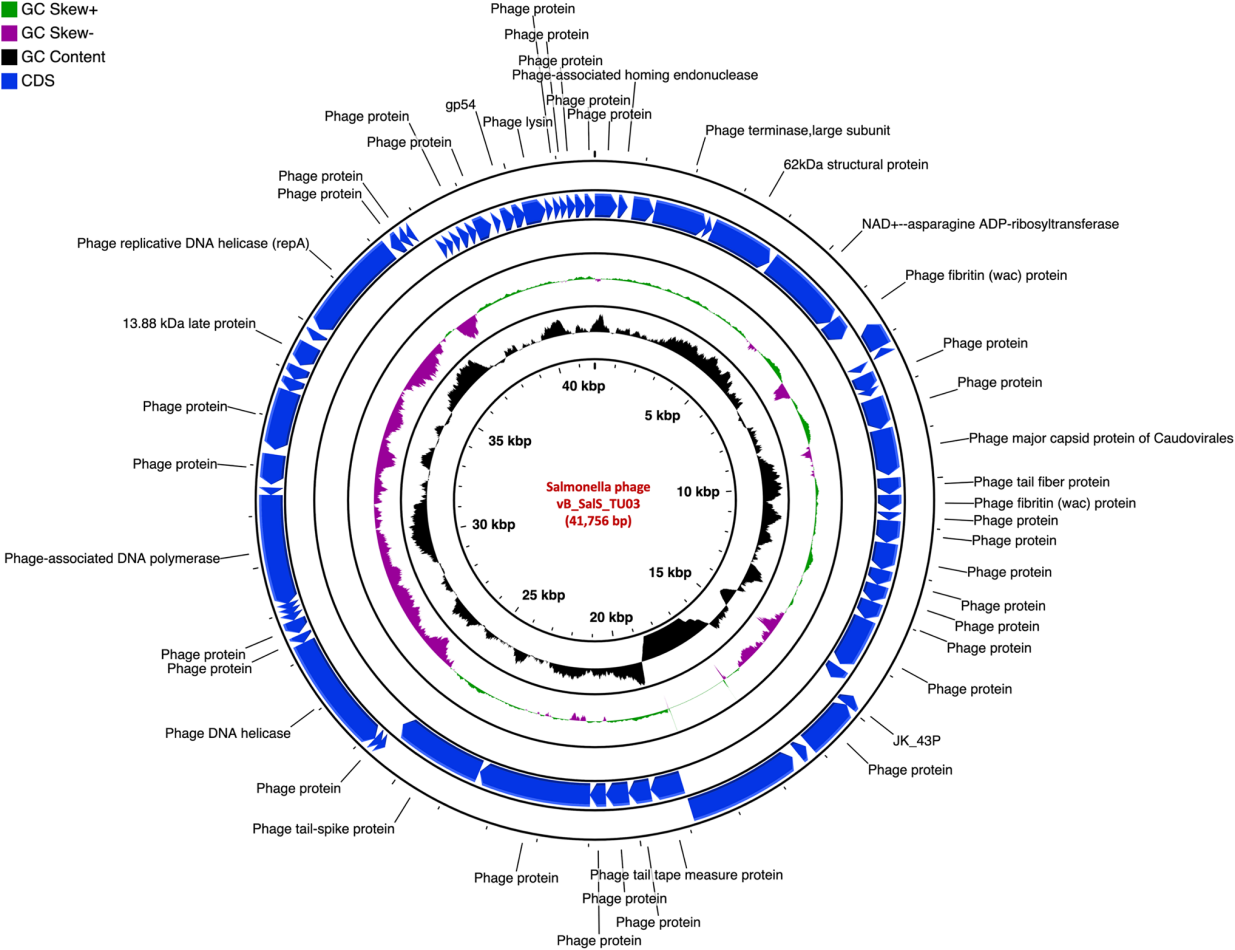
Genome organization of Salmonella phage vB_SalS_TU03 targeting multidrug resistant *Salmonella enterica*. clinical isolate. Predicted coding regions are shown by arrows indicating the direction of the transcription

The genome of vB_EcoM_TU01 was 169,046 bp with a G + C content of 37.42% [lower than that of its host *E. coli* (~ 50.6%)] encoding 286 proteins (Fig. 2). The average length of genes was 566 bp with a CDS coverage of 95.9%. Furthermore, vB_EcoM_TU01encoded 2 transfer-RNAs (tRNA) (tRNA-Met-CAT and tRNA-Arg-TCT). Regarding the gene function, 83.2% (238/286), were functional of which 5.6% (16/286) had a Gene Ontology (GO) assigned function, and the remaining 16.8% (48/286) were hypothetical. Similarly, the genome of vB_KpnM_TU02 was 166,230 bp with a G + C content of 38.34% [lower than that of its host *K. pneumoniae* (~ 57%)] and encoded 294 proteins (Fig. 3). The average gene size in vB_KpnM_TU02 was 540 bp with a CDS coverage of 95.6%. The phage vB_KpnM_TU02 also encoded 15 tRNAs (tRNA-Thr-TGT, tRNA-Leu-TAA, tRNA-Arg-TCT, tRNA-Met-CAT, tRNA-Pro-TGG, tRNA-Gly-TCC, tRNA-Trp-CCA, tRNA-Ile-GAT, tRNA-Ser-TGA, tRNA-His-GTG, tRNA-Gln-TTG, tRNA-Met-CAT, tRNA-Asn-GTT, tRNA-Lys-TTT and tRNA-Tyr-GTA). Out of 294 encoded proteins, 110 (37.4%) were functional, and 184 (62.6%) were hypothetical, whereas only 11 (3.7%) encoded proteins had GO assigned function. Further, the genome of vB_SalS_TU03 was 41,756 bp with a G + C content of 47.06% [slightly lower than that of its host *Salmonella* (~ 52.2%)] and encoded 71 proteins (Fig. 4). The average gene size in vB_SalS_TU03 was 562 bp with a CDS coverage of 95.7%. Out of 71 encoded proteins, 45 (63.4%) aligned with the functional protein whereas 26 (36.6%) were hypothetical. Only 2 out of 71 (2.8%) encoded proteins had GO assigned function.

Although the functions of tRNA in phages remain elusive, it is believed that more tRNA corresponds to increased virulence of the phage as it facilitates a more robust integration of the phages (Bailly-Bechet et al. 2007; Almeida et al. 2022). Since two of our phages encoded multiple tRNAs, it is more likely that these phages are virulent (lytic) and thus more suitable for therapeutic purposes. The ‘functional’ proteins include proteins involved in DNA packaging, transcription, replication, regulation, lysis and structural proteins whereas ‘hypothetical’ proteins are coding DNA sequences (CDS) with unknown functions. All the three phage genomes were free from genes encoding known toxins, antibiotic resistant genes (ARGs), virulent factors (VFs) of bacterial origin and lysogenic markers such as integrase, recombinase, repressor/anti-repressor protein, and excisionase. However, the in silico tool we used (phageAI) only categorized vB_EcoM_TU01 and vB_KpnM_02 as virulent/lytic with high confidence (96.34% and 99.27%, respectively), whereas vB_SalS_TU03 was tagged as temperate/lysogenic with a low confidence of 57%. The substantial number of hypothetical proteins in all phages clearly indicates that phages carry numerous genes that are yet to be characterized, and whose function is yet to be understood. The detailed information about the genomes of all three phages and their respective lifestyle is summarized in Table 2. These results suggest that vB_EcoM_TU01 and vB_KpnM_02 could potentially be used as therapeutic phages against multidrug resistant *E. coli* and *K. pneumoniae,* whereas vB_SalS_TU03 would less likely succeed in lysing its host as it may switch to lysogenic lifestyle and incorporate in the host genome as a prophage. Since prophages play a catalytic role in disease modulation (Nepal et al. 2022) and are known to carry genes increasing bacterial fitness which could be detrimental to humans (Balcazar 2014; Helbin et al. 2012; Khalil et al. 2016; Kondo et al. 2021; Nepal et al. 2021), such phages are not suitable for phage therapy.

**Fig. 5:**
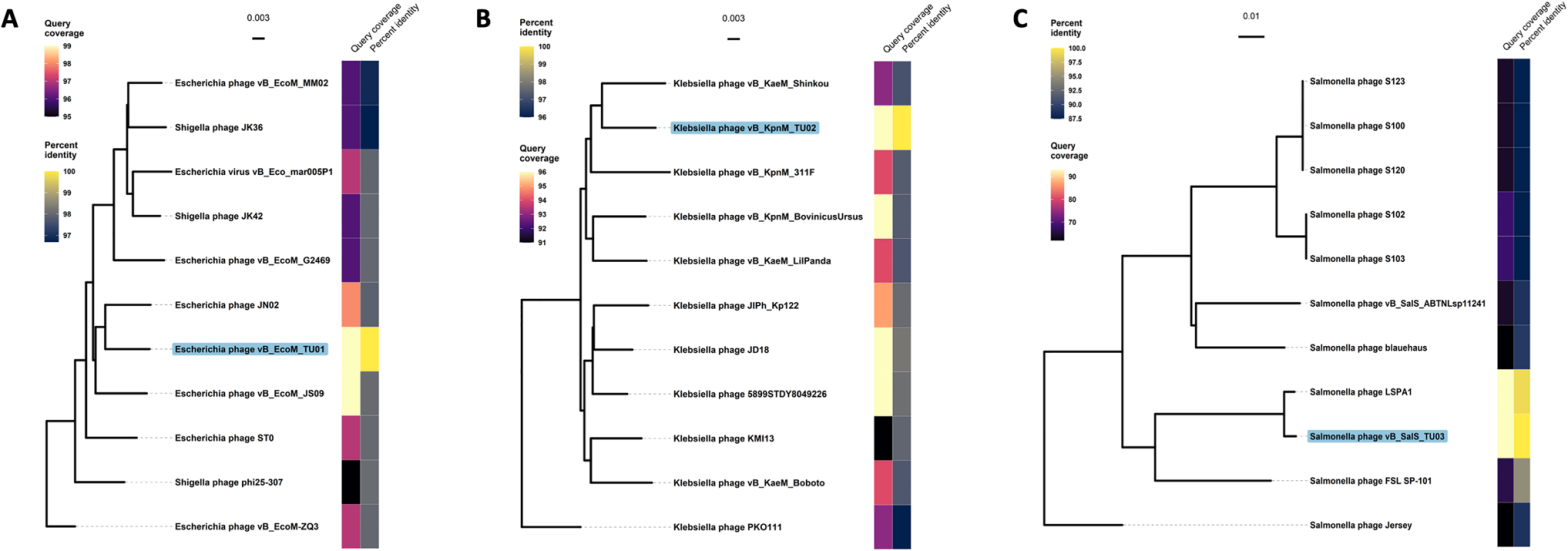
Phylogenetic relatedness of Escherichia phage vB_EcoM_TU01 (**A**), Klebsiella phage vB_KpnM_TU02 (**B**) and Salmonella phage vB_SalS_TU03 (**C**) against most common phage hits (*N* = 10) in the NCBI database. The phylogenetic tree was constructed using neighbour-joining method

Further, comparing the phage genome in the NCBI database using nucleotide BLAST (nBLAST) revealed that the phage vB_EcoM_TU01 was closely related to a T4-like lytic Escherichia phage vB_EcoM_JS09 (NCBI accession = KF582788, query coverage = 99%, per cent identity = 98.04%) isolated in China from the sewage of a swine factory. Similarly, phage vB_KpnM_TU01 was similar to a lytic Klebsiella phage JD18 (NCBI accession = KT239446, query coverage = 96%, per cent identity = 97.89%) isolated in China. Further, phage vB_SalS_TU03 was closest to lytic Salmonella phage LSPA1 (NCBI accession = KM272358, query coverage = 93%, per cent identity = 99.17%) isolated in China from a hospital sewage (Zeng et al. 2015). These analyses indicate that our phages were novel, but highly similar to the phages isolated in neighbouring China around the same time and might have a very similar host range. Phylogenetic relatedness of all three phages against ten most common phages and their per cent identity is elaborated in Fig. 5. It is noted that, among ten most common hits, phylogenetics reveal that vB_EcoM_TU01 is also closely related to *Shigella* phages (also an *Enterobacteriaceae*). Although more study is required, we can arbitrarily predict that phages isolated against different genus of bacteria have higher degree of similarity between them. This may explain (although not studied in this research) why some phages are polyvalent (showing inter-genus or even inter-order infectivity) and show expansive host spectrum (Gambino et al. 2020; Hamdi et al. 2017; Sui et al. 2021; Yu et al. 2016). This property thus holds immense applicability if further study is performed to determine the mechanism of phage infection and identify the factors/proteins/enzymes that determine phage-bacteria specificity.

## Conclusion

Three phages infecting multidrug-resistant *E. coli, K. pneumoniae and S. enterica* were isolated, sequenced and banked. Genome analysis indicated that two of them (Escherichia phage vB_EcoM_TU01 and Klebsiella phage vB_KpnP_TU02) were strictly lytic and free from integrases, virulence factors, toxins, and antimicrobial resistance genes. Although additional studies are required, the genomic features of these phages provide valuable insights into the possibility of using natural phages as biocontrol agents against multidrug resistant human pathogens.

## Supplementary Information

Below is the link to the electronic supplementary material.

Supplementary file1 (DOCX 1234 kb)

## Data Availability

The annotated genome assembly of Escherichia phage vB_EcoM_TU01, Klebsiella phage vB_KpnP_TU02, Salmonella virus vB_SalS_TU03 is available through GenBank accession MZ560701, MZ560702 and MZ560703, respectively. In addition, *fastq* file pertaining to raw sequence data is deposited at NCBI and is available through BioProject accession PRJNA383466 and Sequence Read Archive (SRA) identifiers SRR5460626, SRR5460625, SRR5460624, respectively.

## Abbreviations

MDR: Multidrug resistance
DLAA: Double layer agar assay
PCR: Polymerase chain reaction
CDS: Coding DNA sequence
tRNA: Transfer RNA
ARG: Antibiotic resistant gene
PATRIC: Pathosystems resource integration center
NCBI: National center for biotechnology information
TEM: Transmission electron microscopy
dsDNA: Double-strained DNA
GO: Gene ontology
G+C: Guanine and cytosine
