# Supplementary material for: Genomic characterization of three bacteriophages targeting multidrug resistant clinical isolates of Escherichia, Klebsiella and Salmonella
Source: Arch Microbiol. 2022 May 19;204(6):334. doi: 10.1007/s00203-022-02948-0 (PMC9117343; doi:10.1007/s00203-022-02948-0)
Supplement: Supplementary file 1 — Supplementary file1 (DOCX 1234 kb) [file 203_2022_2948_MOESM1_ESM.docx]

### Supplementary Figure S1 |

Bioanalyser profile of DNA library loaded in Agilient DNA HS chip. The mean sizes of the fragmented libraries (gDNA) of Escherichia phage vB_EcoM_TU01 (A), Klebsiella phage vB_KpnM_TU02 (B), and Salmonella phage vB_SalS_TU03 (C) were 630 bp, 616 bp and 675 bp respectively. (D) Genomic DNA of isolated phages. Marker = Hind III DNA ladder, Lane 1 = Escherichia phage vB_EcoM_TU01, Lane 2 = Klebsiella phage vB_KpnM_TU02, Lane 3 = Salmonella phage vB_SalS_TU03. The numbers on the bottom indicate concentration of genomic DNA in ng/µl as indicated by Qubit 2.0 fluorometer. (E) Average gene length of isolated phages.


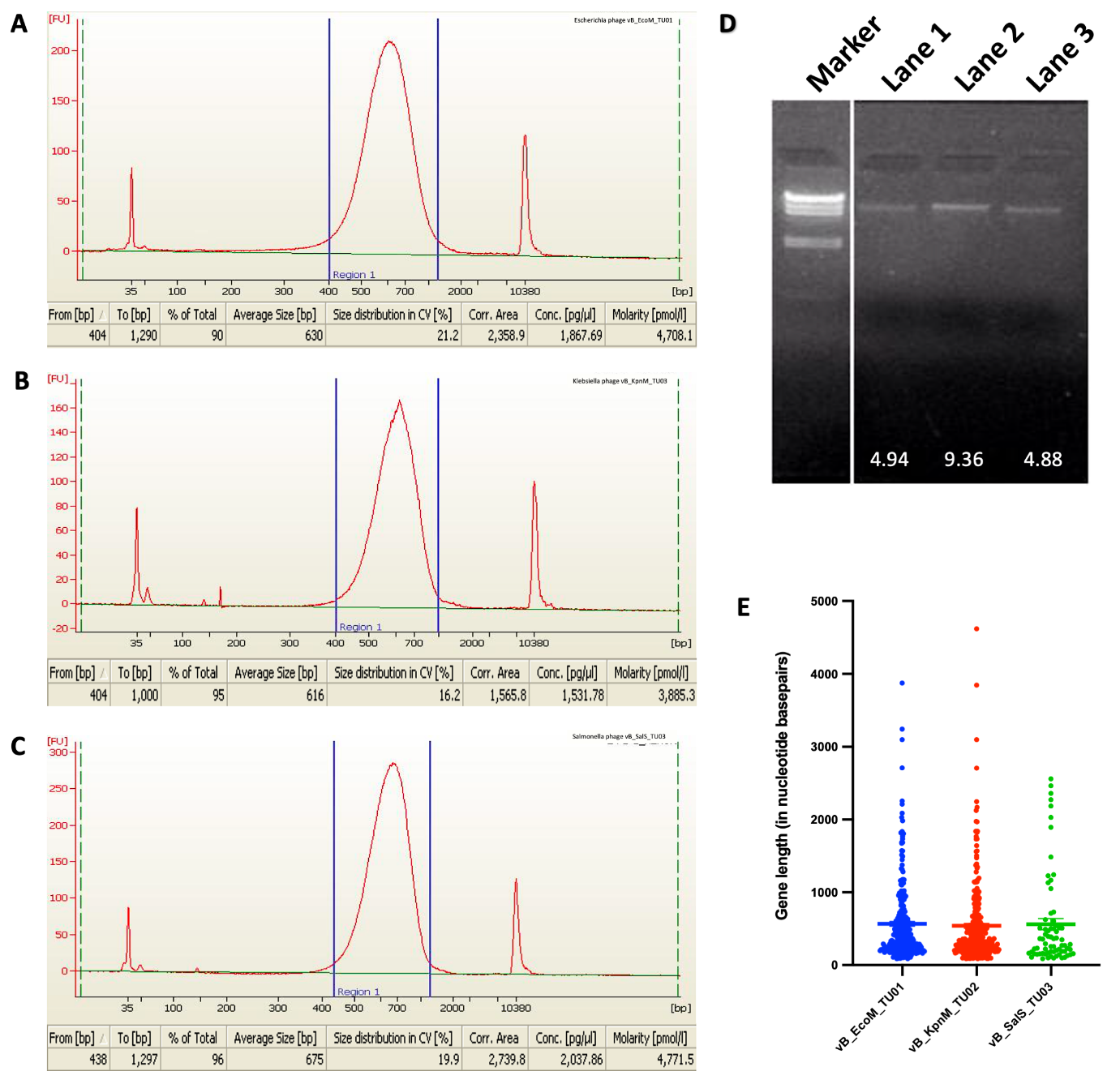


### Supplementary Table S1 |

| Supplementary Table S1 \| Antibiotic susceptibility testing (AST) / Anti-biogram assay of bacterial host strains used for phage isolation using Kirby-Bauer disc diffusion method. | | | | | | | | | | | | | |
| --- | --- | --- | --- | --- | --- | --- | --- | --- | --- | --- | --- | --- | --- |
| **SN** | **Antibiotics**  **Bacteria strains** | **GEN 10** | **MRP 10** | **PIT**  **100/10** | **VA 30** | **NA30** | **AMP 10** | **AK 30** | **OF 5** | **PI100** | **CTX 30** | **MET 5** | **MDR**  **Status** |
| 4 | *Escherichia coli* | **16** | **0** | **18** | **NA** | **0** | **0** | **9** | **0** | **0** | **0** | **NA** | **+** |
| 19 | *Klebsiella pneumoniae* | **0** | **16** | **0** | **NA** | **0** | **0** | **0** | **12** | **0** | **0** | **NA** | **+** |
| 24 | *Salmonella enterica* (NARS) | **22** | **24** | **22** | **NA** | **10** | **9** | **22** | **22** | **15** | **25** | **NA** | **+** |
| Color codes Resistant, Intermediate, Sensitive, Not tested or reference data not available. Numerical value inside differently colored boxes represents bacterial lysis zone in ‘mm’, NA represents ‘not tested’ and or reference data ‘not available’. | | | | | | | | | | | | | |
